# Correction: Characterization of the murine spine for spaceflight studies

**DOI:** 10.1371/journal.pone.0338055

**Published:** 2025-12-05

**Authors:** The PLOS One Staff

## Notice of Republication

An incomplete, earlier version of this article was published in error. The publisher apologises for the error. This article was republished on November 15^th^ 2025, to correct for this error. Please download the article again to view the correct version. The originally published, uncorrected article and the republished, corrected article are provided here for reference.

## Supporting information

S1 File Originally published, uncorrected article. (PDF)

S2 File Republished, corrected article. (PDF)
